# Understanding the role of fear of missing out and deficient self-regulation in sharing of deepfakes on social media: Evidence from eight countries

**DOI:** 10.3389/fpsyg.2023.1127507

**Published:** 2023-03-07

**Authors:** Saifuddin Ahmed, Sheryl Wei Ting Ng, Adeline Wei Ting Bee

**Affiliations:** ^1^Wee Kim Wee School for Communication and Information, Nanyang Technological University, Singapore, Singapore; ^2^Department of Communications and New Media, National University of Singapore, Singapore, Singapore

**Keywords:** deepfakes, disinformation, FOMO, self-regulation, cognitive ability, sharing, self control, Asia

## Abstract

Deepfakes are a troubling form of disinformation that has been drawing increasing attention. Yet, there remains a lack of psychological explanations for deepfake sharing behavior and an absence of research knowledge in non-Western contexts where public knowledge of deepfakes is limited. We conduct a cross-national survey study in eight countries to examine the role of fear of missing out (FOMO), deficient self-regulation (DSR), and cognitive ability in deepfake sharing behavior. Results are drawn from a comparative survey in seven South Asian contexts (China, Indonesia, Malaysia, Philippines, Singapore, Thailand, and Vietnam) and compare these findings to the United States, where discussions about deepfakes have been most relevant. Overall, the results suggest that those who perceive the deepfakes to be accurate are more likely to share them on social media. Furthermore, in all countries, sharing is also driven by the social-psychological trait – FOMO. DSR of social media use was also found to be a critical factor in explaining deepfake sharing. It is also observed that individuals with low cognitive ability are more likely to share deepfakes. However, we also find that the effects of DSR on social media and FOMO are not contingent upon users’ cognitive ability. The results of this study contribute to strategies to limit deepfakes propagation on social media.

## 1. Introduction

Experts have recently warned against the dangers of deepfakes, a form of disinformation created by artificial intelligence. Specifically, deepfakes are highly realistic but synthetically generated video or audio representations of individuals created using artificial intelligence (Westerlund, 2019). They are often more striking, persuasive, and deceptive compared to text-based disinformation (Hameleers et al., 2020). Therefore, the potential dangers of deepfakes have drawn significant academic attention, with several scholars studying public engagement with deepfakes and their consequences (Brooks, 2021; Ahmed, 2021a).

However, there is little evidence on why users share deepfakes on social media. Some studies suggest that users with high political interests and those with low cognitive ability are more likely to share deepfakes (Ahmed, 2021b). Still, there is a lack of psychological explanations for this behavior. For instance, while some studies have explored the role of cognitive ability in reducing the spread of general misinformation (Apuke et al., 2022), there is a lack of research on its effect on deepfake sharing behavior.

Moreover, most current research on deepfakes is based in Western democratic contexts where the general awareness about deepfakes may be higher because they have been featured more heavily in the public discourse, like in the United States. However, it is unclear how these findings would apply in non-Western contexts where public knowledge of deepfakes is limited.

To gain a more complete understanding of why users share deepfakes on social media, it is necessary to examine the role of psychological traits and cognitive ability in deepfake sharing behavior across multiple contexts. More precisely, we focus on a set of psychological (e.g., fear of missing out) and cognitive factors (e.g., cognitive ability) that can help explain deepfake sharing on social media. Further, this study presents the results of a cross-national comparative survey on deepfake sharing behavior in seven South Asian contexts (China, Indonesia, Malaysia, Philippines, Singapore, Thailand, and Vietnam) and compares these findings to the United States, where discussions about deepfakes have been most relevant. The cross-national design of the study increases the generalizability of the findings. The specific goals of the study are outlined below.

In the first step, we examine the role of fear of missing out (FOMO) in deepfake sharing behavior. FOMO is the psychological anxiety that one might be left out of exciting exchanges in their social circles (Przybylski et al., 2013). Some scholars found that FOMO is positively associated with sharing fake news online (Talwar et al., 2019), while others have found it insignificant in predicting fake news sharing (Balakrishnan et al., 2021). These conflicting results highlight the need to clarify the influence of FOMO on misinformation sharing. Nevertheless, regarding deepfakes specifically, FOMO has been found to positively predict intentional deepfake sharing (Ahmed, 2022). However, Ahmed (2022) focused on intentional sharing behavior in two technologically advanced contexts: United States and Singapore. It is unclear whether such findings would be replicated in the less technologically advanced countries we study in this paper. However, if participants in these countries emulate the same processes as social media users in the United States and Singapore, we would expect levels of FOMO to be associated with deepfake sharing behavior. Given the role of FOMO in existing literature, we hypothesize that *FOMO will be positively associated with the sharing of deepfakes* (H1).

Next, this study evaluates the effect of self-regulation on deepfake sharing behavior. Self-regulation is “the process of self-control through the subfunctions of self-monitoring, judgmental process, and self-reaction” (LaRose et al., 2003, p. 232). With sufficient self-regulation, individuals can modulate their behaviors through self-observation. Conversely, deficient self-regulation (DSR), when conscious self-control is weakened (LaRose et al., 2003), manifests in behavioral addictions, such as an addiction to the internet, through a lack of control over impulses (Vally, 2021). Prior studies have reported that DSR significantly predicted unverified information sharing (Islam et al., 2020). DSR is also associated with social media fatigue (Islam et al., 2020; Vally, 2021). In turn, social media fatigue is positively associated with sharing fake news online (Talwar et al., 2019). Hence, we hypothesize that *DSR will be positively associated with the sharing of deepfakes* (H2).

It is also essential to investigate the role of cognitive ability in the sharing of deepfakes as it can provide insight into how individuals make decisions about sharing potentially harmful content. Cognitive ability, which refers to an individual’s mental capacity for problem-solving, decision-making, and learning, can influence how individuals process information and make decisions. An aspect of cognitive ability that is vital to engagement with deepfakes is “the ability or the motivation to think analytically” (Ahmed, 2021b, p. 3). Prior research on the association of cognitive ability with deepfake perception has reported that individuals with higher cognitive ability are less likely to engage in deepfake sharing because they have better discernment and decision-making abilities (Ahmed, 2021b). This may be because individuals with high cognitive ability are known to make sound judgments and are inclined toward problem-solving (Apuke et al., 2022). As such, individuals might perform better at tasks that require reasoning and assessment—such as discerning falsehoods from the truth (Nurse et al., 2021). Although high cognitive ability does not suggest that an individual is infallible to misinformation (Apuke et al., 2022), cognitive ability appears to confer some advantages for navigating misinformation. Given the robustness of cognitive ability in safeguarding users in misinformation engagement, we hypothesize that *cognitive ability will be negatively associated with the sharing of deepfakes* (H3).

Finally, given that cognitive ability can influence deepfake sharing, we also explore whether the effects of FOMO and DSR are contingent upon individuals’ cognitive ability. We anticipate that cognitive ability might act as a buffer against individual traits such as FOMO and DSR in deepfake sharing. Therefore, we pose a research question: *how would cognitive ability moderate the association between (a) FOMO and (b) DSR and the sharin*g *of deepfakes* (RQ1)?

Investigating the moderating effect of cognitive ability on the relationship between FOMO, DSR, and deepfake sharing can provide insight into how individuals make decisions about sharing potentially harmful content. Such a study can also inform interventions and strategies to reduce the spread of harmful deepfake content. Therefore, we report a cross-national comparative study that uses online panel survey data from eight countries to test the relationships between FOMO, DSR, cognitive ability, and deepfake sharing. We also tested for the moderating role of cognitive ability in the relationships mentioned above. Overall, this study contributes to the growing literature on user engagement with disinformation and will help us understand the critical psychological underpinnings of deepfake sharing.

## 2. Materials and methods

### 2.1. Participants

We contracted Qualtrics LLC, a survey research firm agency, to conduct surveys in eight countries, including the United States, China, Singapore, Indonesia, Malaysia, Philippines, Thailand, and Vietnam. We used a quota sampling approach to match the sample to population parameters focusing on age and gender quotas. This was done to generalize our findings to the national adult population. The surveys were conducted concurrently in June 2022 and were translated into national languages. We gathered 1,008 participants (on average) per country – United States (*N* = 1,010), China (*N* = 1,010), Singapore (*N* = 1,008), Indonesia (*N* = 1,010), Malaysia (*N* = 1,002), Philippines (*N* = 1,010), Thailand (*N* = 1,010), and Vietnam (*N* = 1,010). The study was approved by the institutional review board at Nanyang Technological University.

### 2.2. Measurements

*Deepfake sharing* was measured using four deepfake video stimuli (see Supplementary Table A1). The deepfakes were chosen based on their virality on social media. They included a mix of political (e.g., Mark Zuckerberg and Vladimir Putin) and entertainment deepfakes (e.g., Tom Cruise and Kim Kardashian). This approach enhances the external validity of our design. Moreover, other studies have also used some of these deepfakes (see Cochran and Napshin, 2021; Ahmed, 2021a).

For each deepfake video, we ensured that participants were able to play it in full-screen mode. We also asked participants if they could play the video file and successfully watched the video. We then asked how likely they were to share that video on social media with others. Participants responded on a 5-point scale ranging from 1 = extremely to 5 = not at all. We then reverse-coded and averaged their responses across the four stimuli. A higher score represents greater sharing.

*Perceived accuracy* was measured using the same four deepfake stimuli. Since perceived accuracy has been found to closely relate to sharing disinformation (Ahmed, 2021a; t'Serstevens et al., 2022), we have included this as a critical covariate in our analyses. For each deepfake, we asked participants how accurate was the central claim presented in the video (e.g., for the Mark Zuckerberg deepfake we asked, “how accurate is the claim that Mark Zuckerberg said whoever controls the data, controls the future”). Participants responded on a 5-point scale ranging from 1 = not at all accurate, to 5 = extremely accurate. The response for the perceived accuracy of four deepfakes was averaged to create a scale of perceived accuracy.

*Self-regulation (deficient)* was measured using a five-item scale adapted from LaRose and Eastin (2004). Items included “I sometimes try to hide how much time I spend on social media from my family or friends,” and “I feel my social media use is out of control.” among others. Participants responded on a 5-point scale ranging from 1 = strongly disagree to 5 = strongly agree. The items were averaged to create an index of DSR.

*Fear of missing out* was measured using a previously validated 10-item scale (Przybylski et al., 2013). Example items include “I get worried when I find out my friends are having fun without me,” “It bothers me when I miss an opportunity to meet up with friends.” Participants responded on a 5-point scale ranging from 1 = not at all true of me and 5 = extremely true of me. The items were averaged to create an index of FOMO.

*Cognitive ability* was measured by the wordsum test. It includes 10 vocabulary words in which participants are required to find the closest synonym from five options. This test is well-established and has been used widely in literature, including deepfake and fake news studies (Thorndike, 1942; Wechsler, 1958; Ganzach et al., 2019; Ahmed, 2021b).

The descriptive for the variables above can be found in Table 1. All variables met satisfactory reliability in each context (except for cognitive ability in Indonesia, discussed below). See Supplementary Table B1 for details.

**Table 1:** Mean, standard deviation of all variables under study.

	United States	China	Singapore	Indonesia	Malaysia	Philippines	Thailand	Vietnam
Male	46.00%	51.50%	54.90%	50.20%	49.20%	51.30%	52.90%	53.20%
	Mean (SD)	Mean SD)	Mean (SD)	Mean (SD)	Mean (SD)	Mean (SD)	Mean SD)	Mean (SD)
Age	49.0 (17.5)	38.0 (12.7)	43.8 (14.0)	40.3 (13.0)	39.9 (13.7)	37.2 (13.6)	41.6 (13.0)	38.4 (12.8)
Education	5.22 (1.21)	5.72 (0.998)	5.32 (1.22)	5.27 (1.06)	5.77 (1.61)	5.38 (1.11)	5.34 (1.17)	5.55 (0.943)
Income	5.79 (3.76)	6.00 (2.27)	5.47 (2.64)	2.81 (2.39)	4.14 (2.51)	3.26 (2.02)	4.09 (1.91)	6.52 (2.03)
SM news	2.90 (1.49)	3.27 (1.22)	3.17 (1.23)	3.49 (1.21)	3.76 (1.21)	4.17 (0.97)	4.21 (1.03)	3.67 (1.07)
TV news	3.35 (1.35)	3.26 (1.17)	3.11 (1.22)	3.13 (1.24)	3.31 (1.23)	3.94 (1.08)	3.68 (1.22)	3.28 (1.09)
Radio news	2.40 (1.28)	2.46 (1.16)	2.44 (1.22)	2.07 (1.09)	2.64 (1.13)	3.07 (1.22)	2.43 (1.19)	2.32 (1.11)
Print news	2.20 (1.29)	2.12 (1.03)	2.51 (1.31)	2.11 (1.15)	2.63 (1.24)	2.59 (1.23)	2.37 (1.11)	2.33 (1.03)
Perceived accuracy	2.86 (1.04)	3.24 (0.76)	2.41 (0.92)	2.94 (0.8)	2.90 (0.89)	2.81 (0.81)	2.88 (0.87)	2.62 (0.88)
DSR	2.27 (1.16)	2.62 (0.95)	2.43 (1.04)	2.73 (0.88)	2.68 (0.9)	2.61 (0.97)	2.93 (0.90)	2.67 (0.97)
FOMO	2.29 (1.06)	2.53 (0.84)	2.13 (0.95)	2.21 (0.82)	2.22 (0.88)	2.33 (0.88)	2.51 (0.89)	2.44 (0.94)
Cognitive ability	5.43 (2.41)	8.00 (2.09)	5.53 (2.30)	4.78 (1.19)	4.47 (1.79)	6.56 (2.14)	5.81 (1.67)	5.23 (1.86)

### 2.3. Covariates

This study includes several covariates that may influence deepfake sharing behavior. These include demographic variables: (a) age, (b) gender, (c) education, (d) income, (e) social media news consumption, (f) TV news consumption, (g) radio news consumption, and (h) print news consumption (see Table 1).

### 2.4. Analysis

We ran hierarchical regression analyses for the eight countries using SPSS and explored the moderation effects using Hayes (2018) PROCESS macro for SPSS. We also conducted reliability analyses for the key variables and discovered that cognitive ability had low reliability in Indonesia. We thus excluded cognitive ability from our analysis for Indonesia.

Other than running separate regression models for each country, we also ran a pooled regression model. The results are in line with what is presented in the study (see Supplementary Table C1 for details).

## 3. Results

The results of the regression analysis are presented in Table 2. In our preliminary analyses, we found that age was negatively associated with sharing in all countries – United States (β = −0.32, *p* < 0.001), China (β = −0.14, *p* < 0.001), Singapore (β = −0.26, *p* < 0.001), Indonesia (β = −0.18, *p* < 0.001), Malaysia (β = −0.21, *p* < 0.001), Philippines (β = −0.23, *p* < 0.001), and Thailand (β = −0.29, *p* < 0.001), but not Vietnam (β = 0.02, *p* = 0.44). Therefore, suggesting that older adults tend to share less.

**Table 2:** Hierarchical regression analysis predicting deepfakes sharing.

	United States	China	Singapore	Indonesia	Malaysia	Philippines	Thailand	Vietnam
	β	β	β	β	β	β	β	β
Age	−0.32^***^	−0.14^***^	−0.26^***^	−0.18^***^	−0.21^***^	−0.23^***^	−0.29^***^	−0.02
Male	−0.08^***^	−0.05	−0.10^***^	−0.10^***^	−0.11^***^	−0.13^***^	−0.07^*^	−0.12^***^
Education	−0.08^**^	0.04	−0.04	0.06^*^	−0.07^*^	−0.04	−0.03	−0.05
Income	0.05^*^	0.11^**^	−0.01	0.07^*^	−0.02	0.02	0.05	0.00
SM news	0.19^***^	0.14^***^	0.13^***^	0.12^***^	0.04	0.16^***^	−0.01	0.16^***^
TV news	0.02	0.07	−0.02	0.09^*^	−0.04	0.06	0.05	−0.08^*^
Radio news	0.21^***^	0.13^**^	0.19^***^	0.17^***^	0.18^***^	0.08	0.15^***^	0.17^***^
Print news	0.17^***^	0.09^*^	0.11^**^	0.15^***^	0.12^**^	0.12^**^	0.17^***^	0.15^***^
∆*R*^2^	0.42^***^	0.16^***^	0.16^***^	0.23^***^	0.12^***^	0.15^***^	0.19^***^	0.12^***^
*Step 2: Variables of interest*								
Per Accuracy	0.28^***^	0.32^***^	0.31^***^	0.28^***^	0.26^***^	0.18^***^	0.30^***^	0.30^***^
Def self-reg (DSR)	0.07^*^	−0.01	0.16^***^	0.08^**^	0.07^*^	0.05	0.06^*^	−0.00
FOMO	0.25^***^	0.14^***^	0.27^***^	0.18^***^	0.33^***^	0.22^***^	0.28^***^	0.31^***^
Cog ability (CA)	−0.10^***^	−0.05	−0.14^***^	–	−0.11^***^	−0.10^**^	−0.05^*^	−0.16^***^
∆*R*^2^	0.17^***^	0.12^***^	0.32^***^	0.13^***^	0.23^***^	0.11^***^	0.22^***^	0.26^***^
*Step 3: Moderation effects*								
DSR x CA	0.10	−0.07	−0.33^***^	–	0.10	−0.07	0.01	−0.07
FOMO x CA	−0.14	0.26	0.12	–	0.02	0.01	0.19	0.03
∆*R*^2^	0.002	0.003	0.008^***^	–	0.001	0.001	0.002	0.001
Total ∆*R*^2^	0.59	0.28	0.49	0.36	0.35	0.26	0.42	0.38

1. ***p < 0.001; **p < 0.01; *p < 0.05. 2. males = 0, females = 1; 3. cognitive ability was excluded from Indonesia due to low reliability.

We also found a sex effect where males (males = 0, females = 1 in the regression models) were more likely to share in most contexts. The results were significant for the United States (β = −0.08, *p* < 0.001), Singapore (β = −0.10, *p* < 0.001), Indonesia (β = −0.10, *p* < 0.001), Malaysia (β = −0.11, *p* < 0.001), Philippines (β = −0.13, *p* < 0.001), Thailand (β = −0.07, *p* < 0.05), and Vietnam (β = −0.12, *p* < 0.001). The only exception being China (β = −0.05, *p* = 0.11).

Social media news consumption was also a significant predictor of sharing behavior in most countries. Individuals who consumed more social media news were more likely to share in the United States (β = 0.19, *p* < 0.001), China (β = 0.14, *p* < 0.001), Singapore (β = 0.13, *p* < 0.001), Indonesia (β = 0.12, *p* < 0.001), Philippines (β = 0.16, *p* < 0.001), and Vietnam (β = 0.16, *p* < 0.001). The results for Malaysia (β = 0.04, *p* = 0.18) and Thailand (β = −0.01, *p* = 0.76) were statistically insignificant.

We also found that the perceived accuracy of deepfake was a strong and positive predictor in all countries, United States (β = 0.28, *p* < 0.001), China (β = 0.32, *p* < 0.001), Singapore (β = 0.31, *p* < 0.001), Indonesia (β = 0.28, *p* < 0.001), Malaysia (β = 0.26, *p* < 0.001), Philippines (β = 0.18, *p* < 0.001), Thailand (β = 0.30, *p* < 0.001), and Vietnam (β = 0.30, *p* < 0.001). In essence, those who thought the deepfakes were true were more likely to have sharing intentions. These findings are consistent with prior disinformation research (Ahmed, 2021a).

Next, we found strong support for H1. FOMO was positively associated with sharing. This was true for all countries – United States (β = 0.25, *p* < 0.001), China (β = 0.14, *p* < 0.001), Singapore (β = 0.27, *p* < 0.001), Indonesia (β = 0.18, *p* < 0.001), Malaysia (β = 0.33, *p* < 0.001), Philippines (β = 0.22, *p* < 0.001), Thailand (β = 0.28, *p* < 0.001), and Vietnam (β = 0.31, *p* < 0.001).

Next, we found in five of eight countries, namely, the United States (β = 0.07, *p* < 0.05), Singapore (β = 0.16, *p* < 0.001), Indonesia (β = 0.08, *p* < 0.01), Malaysia (β = 0.07, *p* < 0.05), and Thailand (β = 0.06, *p* < 0.05), DSR was positively associated with the sharing of deepfakes. In other words, those who were more impulsive were more likely to share deepfakes. Thus, H2 is supported.

We also found support for H3. Those with higher cognitive ability were less likely to share deepfake. This was true for United States (β = −0.10, *p* < 0.001), Singapore (β = −0.14, *p* < 0.001), Malaysia (β = −0.11, *p* < 0.001), Philippines (β = −0.10, *p* < 0.001), Thailand β = −0.05, *p* < 0.001), and Vietnam (β = −0.16, *p* < 0.001).

As for RQ1, we did not find evidence that cognitive ability moderated the association between (a) FOMO and (b) DSR and deepfake sharing. The only exception was Singapore (β = −0.33, *p* < 0.001); cognitive ability diminished the effects of DSR. In other words, those who were most deficient in self-regulation and had the least cognitive ability were most likely to share deepfakes (Table 2). However, we largely witness that the impact of DSR and FOMO on sharing behavior is not contingent upon the cognitive ability of individuals.

## 4. Discussion

Most studies have investigated social media sharing for general mis- and disinformation. This study is a rare attempt at analyzing sharing associated with deepfakes in eight countries. Overall, the results suggest that those who perceive the deepfakes to be accurate are more likely to share them on social media. Furthermore, in all countries, sharing is also driven by the social-psychological trait – FOMO. DSR of social media use was also found to be a critical factor in explaining sharing of deepfakes. Though, FOMO is a more consistent predictor than DSR. It is also observed that individuals with low cognitive ability are more likely to engage in deepfake sharing. However, we also find that the effects of DSR on social media and FOMO are not contingent upon users’ cognitive ability. In sum, the study identifies critical factors associated with the sharing of deepfakes on social media. The findings are discussed in detail below.

First, the study provides empirical support to the often-discussed relationship between perceived accuracy and sharing of disinformation. In the wider literature, many have questioned the effectiveness of flagged corrections. This is because of the continued influence effect in which people continue to act on their misinformed beliefs even after it has been debunked (Lewandowsky et al., 2012; Ecker and Antonio, 2021). Because the continued influence effect is resilient across situations and people, researchers have generally taken a modest stance towards correcting mis- or disinformation. In view of this debate, the results of this study suggest that targeting accuracy perceptions of deepfakes on social media may still help curtail their propagation. This is in line with a recent study by Ecker and Antonio (2021), which outlined certain conditions for fake news retraction efficacy. They argue that retractions from highly trustworthy and authoritative sources can mitigate the continued influence effect. Similarly, when people are given persuasive reasons to correct their beliefs, they may be less likely to persist in the misbelief and less likely to share the deepfake. Though, given the difference in nature of disinformation (deepfakes vs. other forms), this remains an area worth investigating.

Second, we find strong support for FOMO to be associated with the sharing of deepfakes. Individuals with high levels of FOMO are found to be sensitive and susceptible to distress due to neglect by their social media peers (Beyens et al., 2016). It is possible that such individuals may share deepfakes to gain an opportunity to receive social acceptance and avoid peer neglect on social media. This is in line with Talwar et al.’s (2019) explanation using the self-determination theory. In general, the self-determination theory (SDT) postulates that humans have an innate desire to grow in understanding and make meaning of itself. SDT also posits that people tend to rely on social support. In other words, humans find it difficult to understand themselves without being in relation with others. Therefore, when their sense of relatedness is compromised, they may try to reestablish it by sharing exciting content within their social circle. Moreover, deepfakes are often intriguing, amusing, and provoking and may add to the value of their sharing. Previous evidence also confirms that negative and novel information often spreads more rapidly (Vosoughi et al., 2018) – a characteristic of most deepfakes.

Third, DSR of social media use was positively associated with the sharing of deepfakes in a majority of contexts. The relationship between DSR and sharing can be explained through the fact that when individuals suffer from DSR, they often engage in behaviors that they would not perform if they were self-aware and able to employ a certain level of self-control (LaRose et al., 2003). Here, they may be more likely to share deepfakes. Further, individuals with high DSR may have difficulty managing their emotional reactions to deepfakes. As such, they may feel overwhelmed, and their emotional response could lead them to share on social media, in an attempt to seek support or validation from their social network.

Fourth, individuals with low cognitive ability are vulnerable to deepfake sharing. These results are consistent with existing literature and provide support for the generalizability of the relationships across countries. Individuals with low cognitive ability may have difficulty understanding complex information and applying critical skills in analyzing the authenticity of deepfakes. As such, they may be more susceptible to sharing. Moreover, those with low cognitive ability may also struggle to understand the potential consequences of spreading disinformation.

Finally, while we observe the direct effect of cognitive ability on sharing, we do not observe any moderation effects. In general, the results highlight that individuals with high FOMO and DSR may share, even if they have high cognitive ability and are capable of critically evaluating information, thereby lacking restraint. These patterns confirm that certain social-psychological traits and problematic social media use may override critical thinking. However, future studies could consider using the need for cognition (Cacioppo and Petty, 1982) rather than cognitive ability as a moderator. A higher need for cognition can direct individuals to more cognitive effort toward complex cognitive processes. Further, previous evidence confirms that the successful implementation of self-control requires the availability of limited resources (Schmeichel and Baumeister, 2004). Individuals with a high need for cognition are also found to exhibit greater self-control (Bertrams and Dickhäuser, 2009). Therefore, it may be worthwhile to examine the influence of the need for cognition on not only DSR but also the sharing of deepfakes.

Overall, the factors discussed in this study can help explain why certain individuals contribute to deepfake propagation on social media. To prevent the spread of deepfakes, it is essential to promote healthy self-regulation of social media use and to provide individuals with the tools and skills necessary to manage their excessive use of social media. In addition, promoting interventions that develop the critical skills of individuals with low cognitive ability is also essential. This would safeguard certain groups from spreading disinformation.

Before we conclude, it is important to acknowledge the limitations of this study.

First, the study uses cross-sectional data that limits any causal inferences. While the findings are consistent with previous research, longitudinal study frameworks are necessary to establish causality.

Second, while measuring the effects of social media news use, we did not consider the differences among social media platforms (e.g., WhatsApp vs. Tiktok). Given the differences in affordances across platforms, deepfake sharing behavior may likely vary. Therefore, it is recommended to consider platform differences for more nuanced observations.

Third, our study is based on an online panel of survey respondents. While we use quota sampling strategies to enhance the generalizability of the findings, the results may not be representative of the overall population. The online sample characteristics differ from the general population (see Supplementary Table D1 for distribution). However, our investigation focused on a form of online behavior (sharing); therefore, the representativeness of the findings should be evaluated accordingly.

Fourth, some of our items are single-item (e.g., perceived accuracy), and overall, we use survey methods that are restricted by social desirability biases. Future studies could use unobtrusive data to observe deepfake sharing behavior on social media.

Notwithstanding the limitations, this study offers insights into deepfake propagation on social media by highlighting the role played by perceived accuracy of disinformation, FOMO, DSR of social media use, and cognitive ability. Within this setting, we recommend policymakers that any attempt to reduce the spread of deepfakes on social media should factor in the individual traits of social media users. Intervention programs are less likely to succeed if generalized assumptions are made about social media users, not considering the variance in psychological characteristics and intellectual abilities of audiences.

## Data availability statement

The raw data supporting the conclusions of this article will be made available by the authors, without undue reservation.

## Ethics statement

The studies involving human participants were reviewed and approved by Institutional Review Board, Nanyang Technological University. The patients/participants provided their written informed consent to participate in this study.

## Author contributions

SA designed the study, analyzed the data, and wrote the manuscript. SN wrote the manuscript. AB analyzed the data and wrote the manuscript. All authors approved the submitted version.

## Funding

This work was supported by Nanyang Technological University grant number 21093.

## Conflict of interest

The authors declare that the research was conducted in the absence of any commercial or financial relationships that could be construed as a potential conflict of interest.

## Publisher’s note

All claims expressed in this article are solely those of the authors and do not necessarily represent those of their affiliated organizations, or those of the publisher, the editors and the reviewers. Any product that may be evaluated in this article, or claim that may be made by its manufacturer, is not guaranteed or endorsed by the publisher.
