# Supplementary material for: Understanding the role of fear of missing out and deficient self-regulation in sharing of deepfakes on social media: Evidence from eight countries
Source: Front Psychol. 2023 Mar 7;14:1127507. doi: 10.3389/fpsyg.2023.1127507 (PMC10027756; doi:10.3389/fpsyg.2023.1127507)
Supplement: Supplementary file 1 [file Data_Sheet_1.PDF]

Supplementary Material

Understanding the role of FOMO and deficient self-regulation in sharing of deepfakes on social media: Evidence from eight countries

Saifuddin Ahmed[1*], Sheryl Wei Ting Ng[2], Adeline Bee Wei Ting[1]

[1]Wee Kim Wee School for Communication and Information, Nanyang Technological University, Singapore

[2]Department of Communications and New Media, National University of Singapore, Singapore

*** Correspondence:**
Corresponding Author
sahmed@ntu.edu.sg

1 Supplementary Tables

Supplementary Table A1. Details of deepfakes

Creator	Subject	Link
Bill Posters	Mark Zuckerberg	https://www.instagram.com/p/ByaVigGFP2U/?igshid=MTI5NDc2ZGU=
Bill Posters	Kim Kardashian	https://www.instagram.com/p/ByKg-uKlP4C/?igshid=MTI5NDc2ZGU=
Chris Umé	Tom Cruise	https://www.tiktok.com/@deeptomcruise/video/6932640712861224198?embed_source=70846778%2C120811592%2C120810756%3Bnull%3Bembed_name&is_copy_url=1&is_from_webapp=v1&item_id=6932640712861224198&refer=embed&referer_url=www.independent.co.uk%2Farts-entertainment%2Ffilms%2Fnews%2Ftom-cruise-deepfake-tiktok-video-b1808000.html&referer_video_id=6932640712861224198
Represent Us	Vladimir Putin	https://www.youtube.com/watch?v=sbFHhpYU15w&t=1s

Supplementary Table B1. Cronbach's alpha of all variables under study.

	US	China	Singapore	Indonesia	Malaysia	Philippines	Thailand	Vietnam
**Sharing deepfakes**	.90	.82	.90	.86	.85	.83	.87	.86
**Perceived accuracy**	.74	.57	.75	.65	.65	.58	.70	.71
**Deficient self-regulation**	.90	.87	.91	.82	.86	.87	.84	.87
**FOMO**	.92	.86	.92	.85	.89	.88	.88	.90
**Cognitive ability**	.74	.83	.73	.24	.54	.66	.54	.55

Supplementary Table C1. Pooled regression analyses predicting deepfakes sharing

	All Countries
	β
**Age**	-.25***
**Male**	-.08***
**Education**	-.04***
**Income**	.07***
**SM news**	.12***
**TV news**	.02
**Radio news**	.18***
**Print news**	.09***
ΔR^2	.18***
Step 2: Variables of interest	
**Per Accuracy**	.31***
**Def self-reg (DSR)**	.04***
**FOMO**	.29***
**Cog ability (CA)**	-.06***
ΔR^2	.22***
Step 3: Moderation effects	
**DSR x CA**	-.05
**FOMO x CA**	.01
ΔR^2	.002
**Total** R^2	.40

Note. 1. *** $p < .001$; 2. males = 0, females = 1;3. Indonesia is excluded from the pooled model due to low reliability of cognitive ability.

Supplementary Table D1. Age and sex distribution across countries.

	US	China	Singapore	Indonesia	Malaysia	Philippines	Thailand	Vietnam
**Male %**	49.5	51.3	52.3	50.3	51.4	50.2	48.7	49.9
**Median Age**	39	38	42	30	30	26	40	33

Gender distribution source: https://ourworldindata.org/gender-ratio

Median age source: https://www.worldeconomics.com/Country-Data/
